# Hearing Impairment Is Associated with Smaller Brain Volume in Aging

**DOI:** 10.3389/fnagi.2017.00002

**Published:** 2017-01-20

**Authors:** Stephanie C. Rigters, Daniel Bos, Mick Metselaar, Gennady V. Roshchupkin, Robert J. Baatenburg de Jong, M. Arfan Ikram, Meike W. Vernooij, André Goedegebure

**Affiliations:** ^1^Department of Otorhinolaryngology, Head and Neck Surgery, Erasmus University Medical CenterRotterdam, Netherlands; ^2^Department of Radiology, Erasmus University Medical CenterRotterdam, Netherlands; ^3^Department of Epidemiology, Erasmus University Medical CenterRotterdam, Netherlands; ^4^Department of Neurology, Erasmus University Medical CenterRotterdam, Netherlands

**Keywords:** age-related hearing impairment, pure-tone audiogram, brain MRI, voxel-based analysis, white matter

## Abstract

Although recent studies show that age-related hearing impairment is associated with cerebral changes, data from a population perspective are still lacking. Therefore, we studied the relation between hearing impairment and brain volume in a large elderly cohort. From the population-based Rotterdam Study, 2,908 participants (mean age 65 years, 56% female) underwent a pure-tone audiogram to quantify hearing impairment. By performing MR imaging of the brain we quantified global and regional brain tissue volumes (total brain volume, gray matter volume, white matter (WM) volume, and lobe-specific volumes). We used multiple linear regression models, adjusting for age, sex, head size, time between hearing test and MR imaging, and relevant cognitive and cardiovascular covariates. Furthermore, we performed voxel-based morphometry to explore sub-regional differences. We found that a higher pure-tone threshold was associated with a smaller total brain volume [difference in standardized brain volume per decibel increase in hearing threshold in the age-sex adjusted model: -0.003 (95% confidence interval -0.004; -0.001)]. Specifically, WM volume was associated. Both associations were more pronounced in the lower frequencies. All associations were consistently present in all brain lobes in the lower frequencies and in most lobes in the higher frequencies, and were independent of cognitive function and cardiovascular risk factors. In voxel-based analyses we found associations of hearing impairment with smaller white volumes and some smaller and larger gray volumes, yet these were statistically non-significant. Our findings demonstrate that hearing impairment in elderly is related to smaller total brain volume, independent of cognition and cardiovascular risk factors. This mainly seems to be driven by smaller WM volume, throughout the brain.

## Introduction

Age-related hearing impairment is common in the middle-aged and elderly population (Cruickshanks et al., 2003; Nash et al., 2011; Fischer et al., 2014). This typically starts with loss of hearing sensitivity in the higher frequencies, and progresses over time to the mid and lower frequencies. Simultaneously, the ability to understand speech in noise declines (Gates and Mills, 2005), leading to communication difficulties that have a large impact on a person’s social, psychological, and physical well-being (Dalton et al., 2003).

Primarily, age-related hearing impairment has been described as a condition caused by damage in the peripheral auditory system, in particular the outer hair cells, stria vascularis, or cochlear neurons (Schuknecht and Gacek, 1993). Subsequently, several risk factors for age-related hearing impairment, including noise exposure, cognitive function, and genetic predisposition, have been identified (Gates and Mills, 2005; Humes et al., 2012; Yamasoba et al., 2013).

More recently the focus of research on age-related hearing impairment has expanded toward changes in the central auditory and cerebral system. One of the most commonly studied cerebral regions with regard to hearing impairment is the central auditory pathway, yet the evidence remains inconsistent; some studies demonstrated a relation between hearing impairment and smaller gray matter (GM) volume in this region (Husain et al., 2011; Peelle et al., 2011; Eckert et al., 2012), whereas others found an association with smaller white matter (WM) volume (Chang et al., 2004; Husain et al., 2011), or no association with any brain volume (Profant et al., 2014). One longitudinal study reported an association between hearing impairment and decline in GM volume in the right temporal lobe was found (Lin et al., 2014).

Apart from these contrasting results, an extra consideration which applies to these studies, is that these were performed in relatively small populations who were of various age, and that the effects of important other factors, including cognition, alcohol consumption, and cardiovascular risk factors, were not always taken into account. Hence, larger scale population-based data on elderly with the ability to account for these additional factors are needed. These results will contribute to a further understanding of the link between age-related hearing impairment and structural brain changes.

Therefore, we set out to investigate the association between age-related hearing impairment and morphological brain differences within the large population-based Rotterdam Study.

## Materials and Methods

### Study Design and Subjects

This study was embedded in the Rotterdam Study (Hofman et al., 2015), a population-based prospective cohort study. The study started in 1990 with 7,983 participants that were aged 55 years and older. In 2001, the cohort was extended with 3,011 participants of 55 years and older. A third cohort expansion was performed in 2006 with 3,932 participants aged 45 years and older. Follow-up examinations are performed every 3–4 years in all participants.

From 2005 onward, brain MRI has been incorporated into the core study protocol. Moreover, from 2011 onward, we have been performing hearing assessments in all participants visiting the research center. The current study comprises those participants that underwent both a hearing assessments and brain MRI (*n* = 3,168) until 2014. Ninety-six percent of the MRIs were conducted before or within less than 3 months after hearing assessment [median: 1 month; interquartile range (IQR) -17 months; 2 months].

The Rotterdam Study was approved by the medical ethics committee according to the Population Study Act Rotterdam Study, executed by the Ministry of Health, Welfare, and Sports of the Netherlands. A written informed consent was obtained from all participants.

### Hearing Assessment

All audiometric measurements were performed in a soundproof booth by one trained health care professional. A computer-based clinical audiometry system (Decos Technology Group, version 210.2.6 with AudioNigma interface) and TDH-39 headphones were used.

To determine hearing thresholds in decibel (dB) pure tone audiometry was performed. Thresholds were measured according to the ISO-standard 8253-1 (International Organization for Standardization [ISO], 2010). Air conduction [frequencies 0.25, 0.50, 1, 2, 4, and 8 kilohertz (kHz)] and bone conduction (only two frequencies due to limited time: 0.50 and 4 kHz) were tested for both ears. Masking was done according to the method of Hood (Hood, 1960). Thresholds for the 4 kHz bone conduction were increased with 10 dB afterward based on the discussion on the reference value (Lightfoot and Hughes, 1993; Margolis et al., 2013).

We determined the best hearing ear by taking the average threshold over all frequencies. If both ears were equal, we alternately chose right and left. Furthermore, we determined low (average of 0.25, 0.50, and 1 kHz), speech (average of 0.50, 1, 2, and 4 kHz) and high frequency hearing thresholds (average of 2, 4, and 8 kHz).

We excluded subjects with an air-bone gap of 15 dB or more (*n* = 240), to eliminate clinically relevant conductive hearing loss.

### Brain MRI Acquisition and Processing

**FIGURE 1:**
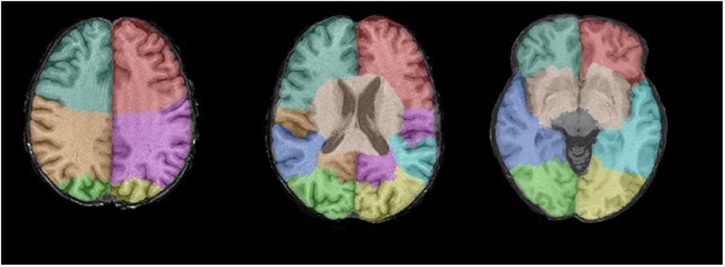
**The template image for the individual lobes as seen from three axial coupes.** Blue–gray = right frontal lobe; Red = left frontal lobe; Brown = right parietal lobe; Purple = left parietal lobe; Green = right occipital lobe; Yellow = left occipital lobe; Blue = right temporal lobe; Turquois = left temporal lobe.

All participants underwent non-contrast enhanced MRI scanning on a 1.5T-scanner (GE Healthcare, Milwaukee, WI, USA). The full MRI protocol has been described extensively before (Ikram et al., 2015). Briefly, the protocol included a T1-weighted sequence, a proton density-weighted sequence, a FLAIR sequence, and a T2^∗^-weighted gradient echo sequence. For all sequences the slice thickness was 1.6 mm (zero-padded to 0.8 mm), except for the FLAIR sequence for which this was 2.5 mm. We used an automated brain tissue classification method, based on a k-nearest-neighbor-classifier algorithm (Vrooman et al., 2007), to quantify the following: total intracranial volume (ICV), total brain volume, GM volume, WM volume, and cerebrospinal fluid volume (in cubic millimeters). To facilitate more regionalized analysis of total brain, GM, and WM volumes, we segmented individual lobes, by non-rigidly registering a template image in which the lobes have been manually outlined (**Figure 1**) (Bokde et al., 2002, 2005).

Voxel based morphometry (VBM) was performed according to an optimized VBM protocol (Good et al., 2001). FSL software (Smith et al., 2004) was used for VBM data processing, all GM and WM density maps were non-linearly registered to the standard ICBM MNI152 GM and WM template (Montreal Neurological Institute) with a 1 mm × 1 mm × 1 mm voxel resolution. Subsequently, a spatial modulation and smoothing procedure with 3 mm (FWHM 8 mm) isotropic Gaussian kernel were applied to all images.

### Other Measurements

We collected detailed information on relevant covariates by interview, physical examination, and blood sampling (Hofman et al., 2015). Assessment of systolic and diastolic blood pressure, Body mass index (BMI), cognition (Mini-Mental State Examination (MMSE)), smoking, and alcohol consumption was done at the research center the day the hearing tests were conducted. Smoking was categorized as never, former, or current. Alcohol consumption was categorized as none-drinker, light consumer (1 unit of alcohol per day for women and 1–2 units of alcohol per day for men), or above average (more than 1 unit of alcohol per day for women and more than 2 units of alcohol per day for men) (Dawson and Room, 2000). Level of education was categorized as having completed primary educational level, secondary educational level, or higher education. Fasting blood samples were obtained and serum total cholesterol and high-density lipoprotein (HDL) cholesterol were measured using an automatic enzymatic procedure (Hitachi analyzer, Roche Diagnostics). Glucose was determined enzymatically by the Hexokinase method. We calculated the cholesterol ratio by the quotient of the total and high density lipoprotein cholesterol. Diabetic mellitus was defined as fasting glucose was 7 mmol/L or more, or non-fasting glucose was 11 mmol/L or more, or subjects used antidiabetic medication, diabetes was stated present.

### Population for Analysis

We performed audiometric measurements and brain MRI in 3,168 subjects. From these, 240 subjects with conductive hearing loss and 20 with an incomplete pure-tone audiogram were excluded, leaving 2,908 subjects for analyses.

### Statistical Analysis

To allow direct comparison of effect estimates, we calculated *Z*-scores for the different brain volumes (total brain volume, GM volume, and WM volume). Missing data on covariates in 305 (10.5%, maximum 7.1% per variable) subjects were entered using multiple imputation (iterations = 5). The difference in standardized brain volume and 95% confidence intervals (CI) were calculated per decibel increase in hearing threshold. This was done for the all tone average, low, speech, and high frequency average. Higher thresholds indicate worse hearing.

Model 1 was adjusted for age (linear and quadratic terms), sex, time between the conduction of MRI and hearing assessment, and ICV. Model 2 was additionally adjusted for MMSE, educational level, systolic and diastolic blood pressure, presence of diabetes mellitus (DM), cholesterol ratio, BMI, smoking, and alcohol consumption.

In the VBM analysis the same linear regression models were fitted with voxel values of GM and WM density as the dependent variable. Statistical significant threshold for family wise error correction was calculated by performing 10.000 random permutation tests, resulting to a *p*-value threshold of 3 × 10^-7^.

Data analysis was done using IBM SPSS Statistics version 21 (IBM, Armonk, NY, USA), and R version 3.1.2 (R Foundation for Statistical Computing, Vienna, Austria).

## Results

### Descriptives

The mean age at the time of the hearing assessment was 64.9 years (SD 7.3) and 56% of the subjects were female. **Table 1** displays all relevant characteristics of the study population.

**Table 1:** Characteristics of the study population (*N* = 2908).

Characteristic	Value
Sex, female	1631 (56.1%)
Age, years	64.9 ± 7.3
Age range, years	52–99
Body mass index, kg/m^2^	27.4 ± 4.2
Education level	
Secondary	1343 (46.1%)
Higher	747 (25.7%)
MMSE score, median (IQR)	29 (27; 29)
Systolic blood pressure, mmHg	138.7 ± 20.4
Diastolic blood pressure, mmHg	82.9 ± 11.0
Diabetes Mellitus, yes	253 (8.7%)
Cholesterol ratio	3.97 ± 1.25
Smoking	
Former	1473 (50.7%)
Current	483 (16.6%)
Alcohol consumption^1^	
Light consumer	2078 (71.5%)
Above average	259 (8.9%)
Intracranial volume, ml	1140.0 ± 115.2
Gray matter, ml	475.5 ± 49.5
White matter, ml	360.7 ± 50.7
Low frequency hearing impairment, dB	14.1 ± 8.3
High frequency hearing impairment, dB	32.4 ± 16.9
Hearing aid users	170 (5.8%)


*^1^Calculated in average grams a day. Light consumer for women is 0–10 g a day, for men 0–20 g a day. Above average for women is more than 10 g a day, for men more than 20 g a day (Dawson and Room, 2000).*

*Unless stated differently values are means (standard deviation) for continuous variables or numbers (percentage) for dichotomous variables. Data represent original data without imputed values. Missing values were present for Body mass index (0,1%), education level (1,0%), MMSE score (0,5%), systolic and diastolic blood pressure (0,5%), cholesterol ratio (2,0%), smoking (0,2%), and alcohol consumption (7,1%).*

*N, Number; IQR, Interquartile range; ml, milliliters; dB, Decibel.*

### Hearing Function and Brain Volumes

**Table 2** shows how hearing impairment is related to brain volume. We found that a higher hearing threshold (i.e., worse hearing) was significantly associated with a smaller brain volume (difference in standardized brain volume per decibel increase in hearing threshold in the age-sex adjusted model: -0.003; CI 95% -0.004; -0.001).

**Table 2:** Associations between auditory function and brain volumes.

	Model 1 (*N* = 2908)		
	
	Total brain volume	Gray matter volume	White matter volume
	
Per dB increase	Difference (CI 95%) *p*-value	Difference (CI 95%) *p*-value	Difference (CI 95%) *p*-value
All Frequencies	-0.003 (-0.004; -0.001) **0.000**	-0.001 (-0.003; 0.001) **0.434**	-0.004 (-0.006; -0.001) **0.003**
Low Frequencies	-0.004 (-0.006; -0.002) **0.000**	-0.001 (-0.003; 0.002) **0.691**	-0.006 (-0.009; 0.000) **0.000**
Speech Frequencies	-0.002 (-0.004; -0.001) **0.000**	-0.001 (-0.003; 0.001) **0.323**	-0.003 (-0.005; -0.001) **0.016**
High Frequencies	-0.001 (-0.002; 0.000) **0.002**	0.000 (-0.002; 0.001) **0.770**	-0.002 (-0.004; 0.000) **0.011**
	
	**Model 2 (*N* = 2908)**		
	
All Frequencies	-0.002 (-0.004; -0.001) **0.001**	-0.001 (-0.003; 0.002) **0.562**	-0.003 (-0.006; -0.001) **0.010**
Low Frequencies	-0.004 (-0.005; -0.002) **0.000**	0.000 (-0.002; 0.003) **0.733**	-0.005 (-0.008; -0.002) **0.001**
Speech Frequencies	-0.002 (-0.003; -0.001) **0.003**	-0.001 (-0.003; 0.001) **0.400**	-0.002 (-0.005; 0.000) **0.046**
High Frequencies	-0.001 (-0.002; 0.000) **0.012**	0.000 (-0.001; 0.001) **0.969**	-0.002 (-0.003; 0.000) **0.026**


*CI, confidence interval; dB, decibel.*

*All frequencies (0.25, 0.50, 1, 2, 4, and 8 kHz); low frequencies (0.25, 0.50, and 1 kHz); speech frequencies (0.50, 1, 2, and 4 kHz); high frequencies (2, 4, and 8 kHz).*

*Outcome indicates the difference (and CI 95%) in standardized brain volume, per decibel increase in hearing threshold (indicating worse hearing). Model 1: Adjusted for age, age^2^, sex, time between MRI and auditory tests, and intracranial volume. Model 2: As model 1, and additionally adjusted for educational level, MMSE score, systolic and diastolic heart rate, BMI, DM, cholesterol ratio, smoking and alcohol consumption. Significant findings (α < 0.05) are shown in bold.*

When brain tissues were analyzed separately, the association with the pure-tone audiogram appeared to be driven by WM volume only (difference in standardized brain volume per decibel increase in hearing threshold -0.004; CI 95% -0.006; -0.001). Above associations were present in all frequencies, however, the effect size was strongest in the lower frequencies.

Adjustment for cardiovascular risk factors, alcohol consumption, education and MMSE score did not alter the associations (**Table 2**, model 2).

### White Matter Volume in the Different Brain Lobes

**FIGURE 2:**
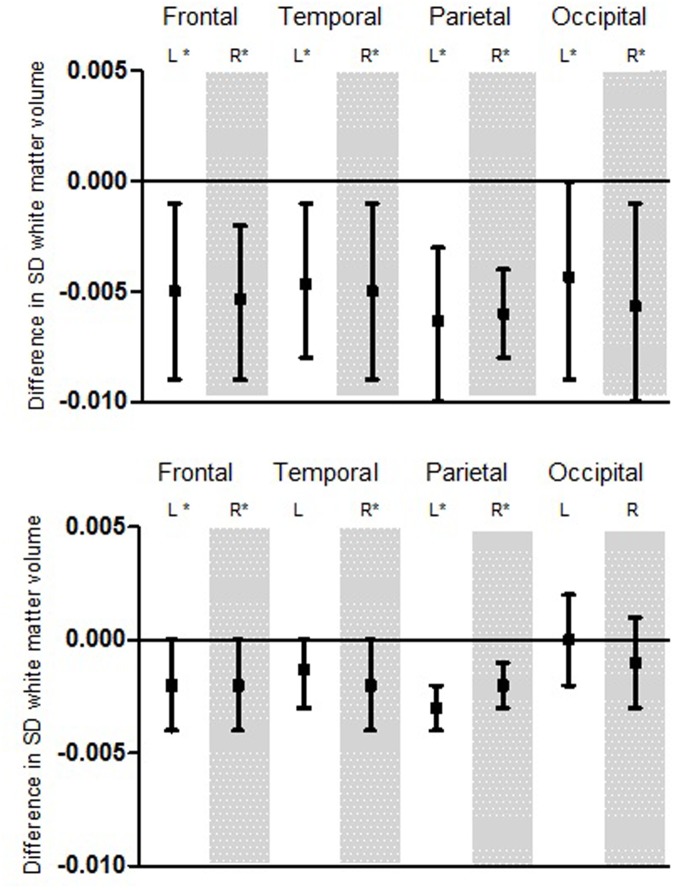
**Association for hearing thresholds and white matter (WM) volume displayed for the different lobes.** First: Variation for low frequency pure-tone threshold. Second: Variation for high frequency pure-tone threshold. Boxplots show the difference in SD WM volume per decibel hearing threshold. Significant findings (α < 0.05) are marked with ^∗^. This model was adjusted for age, age^2^, sex, time between MRI and audio, intracranial volume, educational level, MMSE score, systolic and diastolic heart rate, BMI, DM, cholesterol ratio, smoking and alcohol consumption. L, Left; R, Right; SD, Standard deviation.

Apart from total brain volume, the volumetric parameters of WM in the frontal, temporal, parietal, and occipital lobe were analyzed (**Figure 2**).

We found similar and significant associations among all four brain lobes for low frequency hearing impairment, independently from the side of the hemisphere. Alike the first analysis, a higher hearing threshold (worse hearing) was significantly associated with smaller WM volume.

For high frequency hearing impairment, we also found associations for the frontal, temporal, parietal, and occipital lobe. Although these were not all significant, they point toward a similar effect: a higher hearing threshold (worse hearing) seems to be associated with smaller white volume.

### Voxel-Based Morphometry

After exploring the brain lobes, we conducted exploratory voxel-based analysis to identify if age-related hearing impairment was associated with certain GM and WM regions on voxel level.

**FIGURE 3:**
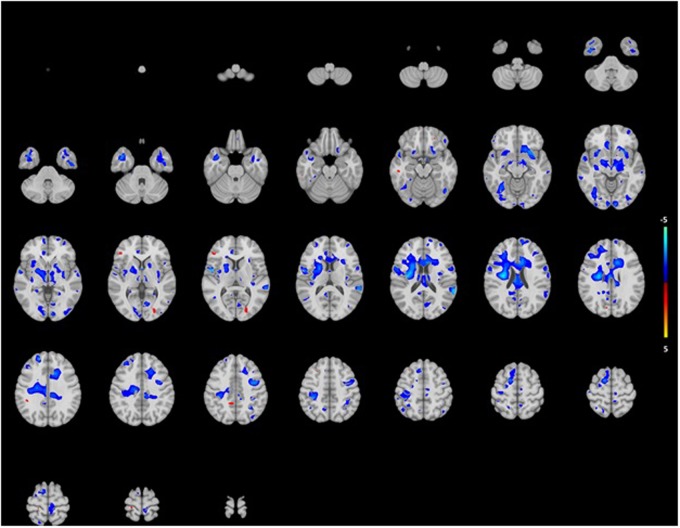
**Projection of voxel based WM areas on axial coupes associated with age-related hearing impairment.** Colors reflect the tendency of the association: blue for a negative direction (decrease of WM), red for a positive direction (increase of WM). See Supplementary Tables for exact outcome per area.

**FIGURE 4:**
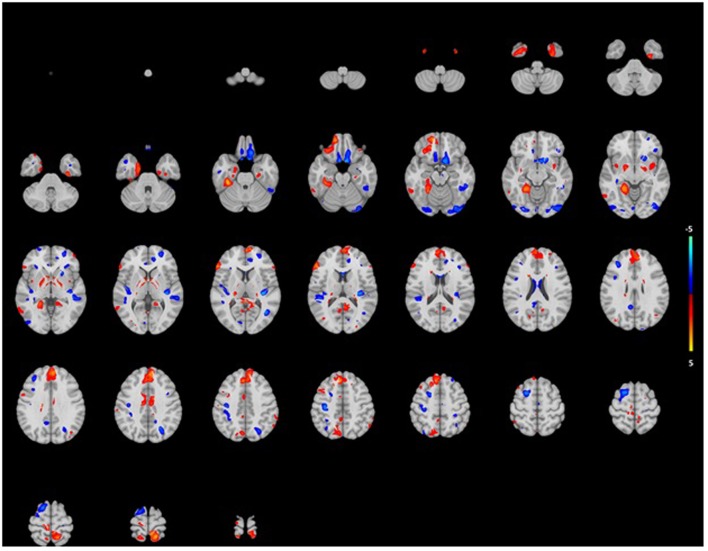
**Projection of voxel based gray matter areas on axial coupes associated with age-related hearing impairment.** Colors reflect the tendency of the association: blue for a negative direction (decrease of gray matter), red for a positive direction (increase of gray matter). See Supplementary Tables for exact outcomes per area.

The analysis showed association for smaller WM in certain areas such as right pre and postcentral gyri, the right insula and the left posterior temporal lobe (**Figure 3**). Mainly, stronger associations were shown in the right hemisphere, while in the posterior temporal lobe this was in the left hemisphere. Again, associations were more pronounced in the lower than the higher frequencies. For GM, we saw association for both smaller and larger volumes in especially the superior frontal gyrus and medial orbital gyrus right, and the superior parietal gyrus left (**Figure 4**). However, none of the voxels reached the multiple-testing correction threshold (**Supplementary Tables 1** and **2**).

## Discussion

In a large sample of community-dwelling elderly persons, we found that hearing impairment was associated with a small total brain volume. These associations were driven by small WM volumes, consistent over all hearing frequencies, and independent of cardiovascular risk factors, alcohol consumption, educational level, and MMSE score. The association between hearing impairment and WM volume was present in all brain lobes. Although not significant, we found mainly an association in our voxel-based analysis in specific areas in the right pre and postcentral gyrus right, the right insula and the left temporal gyrus.

Strengths of this study were the large sample size, the standardized assessment of hearing impairment with pure-tone audiograms, and the automated, volumetric assessment of brain volumes. Moreover, we were able to adjust for multiple determinants that are known to be associated with age-related hearing impairment (Gates and Mills, 2005).

Yet, some limitations of our study should also be addressed. First, although we took into account a large set of potential confounding factors, genetic factors may play a substantial role in the etiology of age-related hearing impairment (Fransen et al., 2015). We were unable to investigate this. Second, due to the cross-sectional design of this study, interpretation of the results with respect to cause and effect is not possible.

### Total Brain, White Matter, and Gray Matter Volume

Although aging causes cortical GM atrophy (Raz et al., 2005) and is also strongly associated with hearing impairment (Gates and Mills, 2005), we found an association between hearing impairment and a small total brain volume independent of age. Additional adjustments for cognition, alcohol consumption, and cardiovascular risk factors did not influence this relation. When investigating brain volume more specifically, the relation between hearing impairment and smaller total brain volume was solely driven by WM volume. This is in line with various other studies that reported age- and sex adjusted associations between hearing impairment and WM volume (Chang et al., 2004; Lin et al., 2008, 2014; Husain et al., 2011). Interestingly, one other study (Lin et al., 2014) did find an significant association between hearing impairment and GM. They found hearing impairment to be related to a decrease of GM volume in specifically the right temporal lobe. We explain these differences in results through differences in study design. By performing a longitudinal study, Lin et al. (2014) could correct for possible intra-individual brain volume differences, thereby eluding the effect of heterogeneity. Unfortunately, this is not to overcome by a cross-sectional design.

Profant et al. (2014) found results similar to ours, as they showed hearing impairment to be not associated with smaller GM volume. This was only influenced by age. Other cross-sectional studies that did find associations with hearing impairment and brain volumes mainly focused on GM in specific brain regions (Eckert et al., 2012), and even found smaller and larger GM volumes contemporaneously in hearing impaired subjects (Husain et al., 2011; Boyen et al., 2013), something we found in our VBM analysis as well.

Several potential mechanisms may be underlying the relation between hearing impairment and brain volume. First, hearing impairment and smaller brain volumes might be the results of mutual risk factors, which is often referred to in the literature as the “common cause” hypothesis (Dawes et al., 2015). This mechanism fits with our findings that suggest a generalized rather than a singular effect in specific brain areas. Such effect might be of microvascular origin, as this is a known process in both cerebral and peripheral auditory systems (Lin et al., 2011).

Second, brain atrophy might induces hearing impairment. This implicates that hearing impairment – as measured by the pure tone audiogram – also reflects certain central auditory changes. It has been generally adopted that the pure tone audiogram only reflects to peripheral auditory function, as it does not require higher central auditory processing (Pickles, 2008). It is difficult to justify that atrophy of the brain would lead to peripheral effects only.

Third, peripheral hearing impairment may induce brain atrophy through neural deprivation of the auditory system by loss of sensory input (Emmorey et al., 2003). This causative effect has already been described in animal studies, although its role is not clear for the aging human auditory system (Ouda et al., 2015). In this case we might expect to find initial atrophy in specific brain lobes that are involved in auditory processing such as the temporal lobe. Although we initially found comparable associations across all brain lobes in our main analysis, our voxel-based analysis revealed stronger associations in certain brain regions.

Poorer hearing was associated with smaller WM volume in the left temporal lobe, which is the location of the auditory cortex and thus involved in high-level auditory processing. The same goes for the right insula, which participates in key auditory processes (Bamiou et al., 2003) and the right pre and post gyri (speech relevant regions). This suggests that volume differences exist in specific brain regions that are more related to the process of hearing impairment than other regions. The associations in the voxel-based analysis were not significant, but we assume this is a power related problem.

It would be interesting to study the effect of hearing aid use in relation to the hypothesis above, as this might reduce the process of neural deprivation. Unfortunately, only 6% (*N* = 170) of the participants in our study wore hearing aids and after excluding them, results did not change.

As has been described previously, different types of age-related hearing impairment arise from different origins (Schuknecht and Gacek, 1993). Strial presbycusis involves atrophy of the stria vascularis especially in the apex and thus affects the lower frequencies. Sensory presbycusis involves loss of outer hair cells especially in the base of the cochlea and thus affects the higher frequencies. As such, stronger associations between WM volume and low frequency thresholds suggest at least some influence of a vascular factor. Although we adjusted for known traditional cardiovascular factors, these factors may not reflect well the more subtle differences in (micro)vascularization that might be involved in the case of age-related hearing impairment. Realistically, the above mentioned hypotheses are not mutually exclusive and could all take (a minor) part in the etiology of age-related decline in hearing and brain morphology.

## Conclusion

Our findings demonstrate that age-related hearing impairment is associated with a smaller total brain volume, specifically WM volume. Furthermore, though the association is found generalized throughout the brain, there is a suggestion of certain brain regions to be more strongly involved. Associations were independent of age, sex, cognitive function, cardiovascular risk factors, and alcohol consumption. Our results contribute to the culminating evidence that age-related hearing impairment and morphological differences in the brain interact. Additional research on this topic is needed to identify the relevant underlying causative mechanisms and possible preventive effects of early treatment, such as timely use of hearing aids.

## Author Contributions

Conception/design of the work: SR, DB, MM, RB, MI, MV, and AG. Acquisition: RB and AG. Analysis: SR, DB, and GR. Interpretation of data: SR, DB, MM, GR, MI, MV, and AG. Drafting the work: SR, DB, MM, MI, MV, and AG. Revising the work critically: SR, DB, MM, GR, RB, MI, MV, and AG. Final approval: SR, DB, MM, GR, RB, MI, MV, and AG.

## Conflict of Interest Statement

The authors declare that the research was conducted in the absence of any commercial or financial relationships that could be construed as a potential conflict of interest.
