# Corrigendum: Hearing Impairment Is Associated with Smaller Brain Volume in Aging

**DOI:** 10.3389/fnagi.2017.00131

**Published:** 2017-05-08

**Authors:** Stephanie C. Rigters, Daniel Bos, Mick Metselaar, Gennady V. Roshchupkin, Robert J. Baatenburg de Jong, M. Arfan Ikram, Meike W. Vernooij, André Goedegebure

**Affiliations:** ^1^Department of Otorhinolaryngology, Head and Neck Surgery, Erasmus University Medical CenterRotterdam, Netherlands; ^2^Department of Radiology, Erasmus University Medical CenterRotterdam, Netherlands; ^3^Department of Epidemiology, Erasmus University Medical CenterRotterdam, Netherlands; ^4^Department of Neurology, Erasmus University Medical CenterRotterdam, Netherlands

**Keywords:** age-related hearing impairment, pure-tone audiogram, brain MRI, voxel-based analysis, white matter

In the original article, Roshchupkin et al. (2016) was not cited in the article. The citation has now been inserted in Materials and Methods, subsection Brain MRI Acquisition and Processing, second paragraph and should read:

Voxel based morphometry (VBM) was performed according to an optimized VBM protocol (Good et al., 2001) and was previously described (Roshchupkin et al., 2016). FSL software (Smith et al., 2004) was used for VBM data processing, all GM and WM density maps were non-linearly registered to the standard ICBM MNI152 GM and WM template (Montreal Neurological Institute) with a 1 mm × 1 mm × 1 mm voxel resolution. Subsequently, a spatial modulation and smoothing procedure with 3 mm (FWHM 8 mm) isotropic Gaussian kernel were applied to all images.

The authors apologize for this error and state that this does not change the scientific conclusions of the article in any way.

## Conflict of interest statement

The authors declare that the research was conducted in the absence of any commercial or financial relationships that could be construed as a potential conflict of interest.
