# Supplementary material for: Hearing Impairment Is Associated with Smaller Brain Volume in Aging
Source: Front Aging Neurosci. 2017 Jan 20;9:2. doi: 10.3389/fnagi.2017.00002 (PMC5247429; doi:10.3389/fnagi.2017.00002)
Supplement: Supplementary file 1 [file Table_1.DOCX]

**Supplementary table 1. Associations of grey matter in voxel-based morphometry with hearing loss over all frequencies.**

| Brain area left hemisphere | p-value | In /  decrease | Brain area right hemisphere | p-value | In/  decrease |
| --- | --- | --- | --- | --- | --- |
| Hippocampus | 1.0 | = | Hippocampus | 0.00241 | + |
| Amygdala | 1.0 | = | Amygdala | 0.00398 | + |
| Anterior temporal lobe, medial part | 0.00534 | + | Anterior temporal lobe, medial part | 0.00062 | + |
| Anterior temporal lobe, lateral part | 1.0 | = | Anterior temporal lobe, lateral part | 1.0 | = |
| Gyri parahippocampalis et ambiens | 0.00465 | + | Gyri parahippocampalis et ambiens | 0.00199 | + |
| Superior temporal gyrus, central part | 8.42e-^05^ | - | Superior temporal gyrus, central part | 0.00347 | - |
| Medial and inferior temporal gyri | 1.0 | = | Medial and inferior temporal gyri | 0.00088 | + |
| Lateral occipitotemporal gyrus (fusiformis) | 0.00063 | + | Lateral occipitotemporal gyrus (fusiformis) | 3.70 e-^05^ | + |
| Insula | 7.74 e-^05^ | -/+ | Insula | 0.00114 | -/+ |
| Lateral remainder of occipital lobe | 0.00071 | - | Lateral remainder of occipital lobe | 0.00138 | -/+ |
| Cingulate gyrus anterior (supragenual) | 0.00269 | -/+ | Cingulate gyrus anterior (supragenual) | 1.0 | = |
| Cingulate gyrus posterior | 0.00220 | + | Cingulate gyrus posterior | 0.00364 | -/+ |
| Middle frontal gyrus | 0.00051 | -/+ | Middle frontal gyrus | 0.00040 | -/+ |
| Posterior temporal lobe | 0.00035 | -/+ | Posterior temporal lobe | 5.13 e-^05^ | -/+ |
| Remainder of parietal lobe (including supramarginal and angular gyrus) | 0.00124 | - | Remainder of parietal lobe (including supramarginal and angular gyrus) | 0.00460 | -/+ |
| Caudate nucleus | 0.00048 | - | Caudate nucleus | 0.00147 | + |
| Nucleus accumbens | 0.00019 | - | Nucleus accumbens | 1.0 | = |
| Putamen | 0.00026 | - | Putamen | 1.0 | = |
| Thalamus | 0.00068 | + | Thalamus | 0.00019 | + |
| Pallidum (globus pallidus) | 0.00155 | + | Pallidum (globus pallidus) | 0.00074 | + |
| Lateral ventricle frontal horn central part and occipital horn | 0.00123 | -/+ | Lateral ventricle frontal horn central part and occipital horn | 0.00026 | -/+ |
| Lateral ventricle temporal horn | 1.0 | = | Lateral ventricle temporal horn | 1.0 | = |
| Precentral gyrus | 0.00525 | + | Precentral gyrus | 0.00032 | -/+ |
| Straight gyrus (gyrus rectus) | 1.71 e-^05^ | - | Straight gyrus (gyrus rectus) | 3.52 e-^05^ | - |
| Anterior orbital gyrus | 1.0 | = | Anterior orbital gyrus | 8.12 e-^05^ | + |
| Inferior frontal gyrus | 0.00663 | - | Inferior frontal gyrus | 0.00030 | + |
| Superior frontal gyrus | 0.00017 | -/+ | Superior frontal gyrus | 4.70 e-^05^ | -/+ |
| Postcentral gyrus | 0.00027 | -/+ | Postcentral gyrus | 0.00064 | -/+ |
| Superior parietal gyrus | 6.38 e-^05^ | -/+ | Superior parietal gyrus | 0.00181 | -/+ |
| Lingual gyrus | 1.0 | = | Lingual gyrus | 0.00013 | + |
| Cuneus | 1.0 | = | Cuneus | 1.0 | = |
| Medial orbital gyrus | 0.00026 | - | Medial orbital gyrus | 2.55 e-^05^ | -/+ |
| Lateral orbital gyrus | 0.00462 | - | Lateral orbital gyrus | 1.0 | = |
| Posterior orbital gyrus | 0.00505 | - | Posterior orbital gyrus | 0.00326 | + |
| Substantia nigra | 0.00431 | - | Substantia nigra | 1.0 | = |
| Subgenual anterior cingulate gyrus | 1.0 | = | Subgenual anterior cingulate gyrus | 0.00636 | - |
| Subcallosal area | 0.00155 | - | Subcallosal area | 0.00470 | - |
| Pre-subgenual anterior cingulate gyrus | 1.0 | = | Pre-subgenual anterior cingulate gyrus | 1.0 | = |
| Superior temporal gyrus, anterior part | 1.0 | = | Superior temporal gyrus, anterior part | 1.0 | = |
| **Other brain area** |  |  |  |  |  |
| Corpus callosum | 0.00010 | -/+ |  |  |  |
| Third ventricle | 0.00634 | + |  |  |  |

*‘*-‘ = decrease in grey matter; ‘+’ = increase in grey matter, ‘-/+’ = both decrease and increase in grey matter, ‘=’ = no change in grey matter.
