# Supplementary material for: Hearing Impairment Is Associated with Smaller Brain Volume in Aging
Source: Front Aging Neurosci. 2017 Jan 20;9:2. doi: 10.3389/fnagi.2017.00002 (PMC5247429; doi:10.3389/fnagi.2017.00002)
Supplement: Supplementary file 2 [file Table_2.DOCX]

**Supplementary table 2. Associations of white matter in voxel-based morphometry with hearing loss over all frequencies.**

| Brain area left hemisphere | p-value | In /  decrease | Brain area right hemisphere | p-value | In/  decrease |
| --- | --- | --- | --- | --- | --- |
| Hippocampus | 0.00933 | - | Hippocampus | 1.0 | = |
| Amygdala | 0.00662 | - | Amygdala | 1.0 | = |
| Anterior temporal lobe, medial part | 0.00724 | - | Anterior temporal lobe, medial part | 0.00038 | - |
| Anterior temporal lobe, lateral part | 1.0 | = | Anterior temporal lobe, lateral part | 0.00074 | - |
| Gyri parahippocampalis et ambiens | 0.00618 | - | Gyri parahippocampalis et ambiens | 1.0 | = |
| Superior temporal gyrus, central part | 0.00397 | - | Superior temporal gyrus, central part | 0.00133 | - |
| Medial and inferior temporal gyri | 0.00174 | - | Medial and inferior temporal gyri | 0.00082 | - |
| Lateral occipitotemporal gyrus (fusiformis) | 0.00719 | - | Lateral occipitotemporal gyrus (fusiformis) | 0.00763 | - |
| Insula | 0.00029 | - | Insula | 4.70 e-^05^ | - |
| Lateral remainder of occipital lobe | 0.00179 | - | Lateral remainder of occipital lobe | 0.00118 | - |
| Cingulate gyrus anterior (supragenual) | 0.00045 | - | Cingulate gyrus anterior (supragenual) | 0.00120 | - |
| Cingulate gyrus posterior | 0.00036 | - | Cingulate gyrus posterior | 0.00020 | - |
| Middle frontal gyrus | 0.00048 | - | Middle frontal gyrus | 0.00025 | -/+ |
| Posterior temporal lobe | 3.57 e-^05^ | - | Posterior temporal lobe | 0.00105 | - |
| Remainder of parietal lobe (including supramarginal and angular gyrus) | 2.12 e-^05^ | - | Remainder of parietal lobe (including supramarginal and angular gyrus) | 0.00079 | - |
| Caudate nucleus | 0.00090 | - | Caudate nucleus | 0.00070 | - |
| Nucleus accumbens | 1.0 | = | Nucleus accumbens | 1.0 | = |
| Putamen | 0.00032 | - | Putamen | 0.00201 | - |
| Thalamus | 0.00167 | - | Thalamus | 0.00038 | - |
| Pallidum (globus pallidus) | 0.00435 | - | Pallidum (globus pallidus) | 0.00145 | - |
| Lateral ventricle frontal horn central part and occipital horn | 0.00092 | - | Lateral ventricle frontal horn central part and occipital horn | 0.00077 | - |
| Lateral ventricle temporal horn | 0.00757 | - | Lateral ventricle temporal horn | 1.0 | = |
| Precentral gyrus | 0.00043 | - | Precentral gyrus | 3.34 e-^05^ | - |
| Straight gyrus (gyrus rectus) | 1.0 | = | Straight gyrus (gyrus rectus) | 1.0 | = |
| Anterior orbital gyrus | 1.0 | = | Anterior orbital gyrus | 1.0 | = |
| Inferior frontal gyrus | 0.00595 | - | Inferior frontal gyrus | 0.00455 | + |
| Superior frontal gyrus | 0.00045 | - | Superior frontal gyrus | 0.00013 | - |
| Postcentral gyrus | 0.00393 | - | Postcentral gyrus | 8.02 e-^05^ | - |
| Superior parietal gyrus | 0.00082 | - | Superior parietal gyrus | 0.00064 | - |
| Lingual gyrus | 0.00065 | - | Lingual gyrus | 0.00057 | - |
| Cuneus | 0.00821 | - | Cuneus | 0.00251 | - |
| Medial orbital gyrus | 0.00152 | - | Medial orbital gyrus | 1.0 | = |
| Lateral orbital gyrus | 0.00685 | - | Lateral orbital gyrus | 1.0 | = |
| Posterior orbital gyrus | 0.00124 | - | Posterior orbital gyrus | 1.0 | = |
| Substantia nigra | 0.00806 | - | Substantia nigra | 1.0 | = |
| Subgenual anterior cingulate gyrus | 0.00212 | - | Subgenual anterior cingulate gyrus | 0.00296 | - |
| Subcallosal area | 0.00181 | - | Subcallosal area | 1.0 | = |
| Pre-subgenual anterior cingulate gyrus | 1.0 | = | Pre-subgenual anterior cingulate gyrus | 0.00778 | - |
| Superior temporal gyrus, anterior part | 1.0 | = | Superior temporal gyrus, anterior part | 0.00122 | - |
| **Other brain area** |  |  |  |  |  |
| Corpus callosum | 0.00027 | - |  |  |  |
| Third ventricle | 0.00404 | - |  |  |  |

*‘*-‘ = decrease in white matter; ‘+’ = increase in white matter, ‘-/+’ = both decrease and increase in white matter, ‘=’ = no change in white matter.
